# Enhancing well-being in higher education: the role of job satisfaction and resilience among ELT instructors

**DOI:** 10.3389/fpsyg.2025.1629498

**Published:** 2025-07-28

**Authors:** Senem Zaimoğlu, Aysun Dağtaş

**Affiliations:** English Translation and Interpreting Department, Çağ University, Mersin, Türkiye

**Keywords:** psychological well-being, job satisfaction, teacher resilience, ELT instructors, higher education

## Abstract

**Introduction:**

As academic demands intensify and institutional pressures increase, university-level English Language Teaching (ELT) instructors face growing risks to their psychological well-being. Understanding the factors that contribute to their well-being is essential for ensuring sustainable professional engagement. This study investigates the role of job satisfaction and teacher resilience in promoting psychological well-being among ELT instructors in higher education settings.

**Methods:**

A quantitative research design was employed, involving 173 ELT instructors working at universities in Türkiye. Data were collected through three validated instruments: the Teacher Job Satisfaction Scale, the Teacher Resilience Scale, and the Psychological Well-Being Scale. Multiple regression analyses were used to explore the predictive relationships among the variables.

**Results:**

Findings revealed that both job satisfaction and resilience significantly and positively predicted psychological well-being. Among the two, job satisfaction emerged as the strongest predictor. In addition, teacher resilience was found to partially mediate the relationship between job satisfaction and psychological well-being.

**Discussion:**

The results highlight the importance of fostering emotionally resilient and professionally satisfied ELT instructors. Institutional policies that enhance autonomy, promote professional development, and support well-being are critical for sustaining mental health and effective teaching performance in demanding higher education contexts.

## Introduction

Teachers’ psychological well-being has emerged as a growing concern in higher education, particularly as faculty are being asked to manage heightened expectations of teaching quality, research output, administrative engagement, and flexibility for evolving digital environments (Skaalvik and Skaalvik, 2017; Mercer and Gregersen, 2020). University-level ELT instructors often face institutional pressures as they work in multilingual, multicultural classrooms and carry intense cognitive, affective, and administrative loads (Dewaele and Li, 2020). Although they play a crucial role in the academic literacy and the development of students’ global competences, the psychological well-being of ELT teachers has received limited attention, especially in higher education contexts. Psychological well-being, as described by Ryff (1989), is a complex construct that includes autonomy, environmental mastery, personal growth, positive relations with others, purpose in life, and self-acceptance. These dimensions go beyond the absence of burnout or distress to capture a more integrated view of teachers’ mental health and job satisfaction (Ryff and Keyes, 1995). In ELT, psychological well-being is important not only for the health and job stress of instructors but also for the quality of instruction and student success (Mercer, 2021; Gao et al., 2025).

Among the numerous psychological and organizational determinants of teachers’ well-being, two have been consistently identified as good predictors: job satisfaction and resilience (Brunetti, 2006; Zee and Koomen, 2016). Job satisfaction is understood as the degree to which teachers indicate being satisfied with their job, determined by factors such as professional autonomy, institutional support, appreciation, and peer relationships (Klassen and Chiu, 2010; Skaalvik and Skaalvik, 2011). Greater job satisfaction has been identified to have a positive impact on mental health, reduced turnover intentions, and enhance teaching motivation (Day and Qing, 2009). Resilience, however, embodies one’s capacity to withstand and recover from stress, adversity, and setbacks (Gu and Day, 2007). For ELT teachers, resilience is important in managing classroom issues like student disengagement, cultural misunderstandings, pressure in performance, and typically limited pedagogical resources (Zarei and Hosseini, 2020). Research has revealed that resilient teachers have a greater ability to maintain emotional balance, accommodate institutional demands, and keep lasting commitment towards being a teacher (Beltman et al., 2011).

While job satisfaction and resilience have been examined in various educational contexts such as elementary school, secondary school, and high school context (Roman-Oertwig, 2004; Demir Polat and İskender, 2018), their interrelation and combined impact on psychological well-being—particularly among ELT teaching professionals in tertiary environments—have not been explored sufficiently. Most studies so far have involved examining K–12 teachers or pre-service teachers, without taking into account the more complex professional ecology of university-level ELT teaching professionals (Bacolod et al., 2024; Benevene et al., 2020a; Ortan et al., 2021). Moreover, ELT teachers usually have to bear additional burdens like overloads of courses, administrative tasks, standardization of curricula requirements, and limited channels of professional advancement—all of which, if not buffered with internal (e.g., resilience) and environmental (e.g., job satisfaction) resources, can erode psychological welfare.

In order to bridge these gaps, the present study explores the predictive functions of job satisfaction and teacher resilience in ELT university teachers’ psychological well-being in Türkiye. It aims to examine the direct effects of both variables on well-being and the mediating role of job satisfaction in the relationship between resilience and psychological well-being. In so doing, this research adds to an increasing body of positive psychology literature on teacher wellbeing, providing evidence of how organizational and individual variables combine to influence the psychological wellbeing of ELT professionals within higher education institutions.

## Literature review

### Teacher job satisfaction

Job satisfaction is widely regarded as a basic component of teachers’ occupational wellbeing and professional commitment. It refers to the extent to which teachers feel satisfaction, appreciation, and support in working contexts (Skaalvik and Skaalvik, 2011). Job satisfaction in higher education institutions is motivated by a variety of interrelated factors, including professional autonomy, recognition, workload balance, collegial relationships, and institutional leadership (Klassen and Chiu, 2010). Content teachers are also more likely to exhibit stronger teaching efficacy, lower emotional exhaustion, and higher organizational commitment (Day and Qing, 2009; Demirtas, 2010; Judge et al., 2001). Thus indirectly, job satisfaction not only contributes to individual psychological health but also school health as a whole by reducing teacher turnover and increasing instructional quality. Teachers who find their profession worthwhile and are well supported tend to become emotionally and mentally committed in their teaching career, hence developing more challenging and effective learning environments (OECD, 2021). Job satisfaction is also crucial for protecting teachers from the detrimental effects of occupational stress and burnout, which are prevalent in difficult academic settings (Carroll et al., 2022). If educators are constantly subjected to discontent—due to such factors as a lack of institutional support, negative expectations, or an excessive burden of administrative tasks—their psychological resilience will deteriorate, ultimately threatening their well-being and performance (Skaalvik and Skaalvik, 2017).

In the profession of ELT, job satisfaction takes particular forms due to the intercultural and multilingual nature of teaching, which is typically combined with limited institutional resources and enormous pressures for academic productivity (Mercer, 2020). Mercer and Gregersen (2020) believe that particularly in ELT settings, where teachers typically struggle with different learner needs, curriculum reform, and multilinguality, job satisfaction can serve as a psychological anchor to facilitate resilience and emotional regulation. Studies of Turkish higher education have reported that, where ELT instructors perceive their contributions to be valued and professional opportunities exist, satisfaction with employment significantly increases (Tayfur et al., 2013). The opposite conditions which were reasons for dissatisfaction proved to be limited administrative support, inelastic curriculum and heavy workloads (Yean et al., 2022). Parallel patterns have also emerged in other environments beyond the national environment. In Japan, for instance, Sato and Kleinsasser (2004) stated that official recognition and cooperation were significant predictors of English teachers’ job satisfaction, while bureaucratic constraint and ill-defined performance expectations did so in the opposite direction. In Middle Eastern societies, Alzaanin (2021) highlighted that the amount of workload and opportunity for professional development were significant predictors of job satisfaction among university EFL instructors. Likewise, in a European large-scale study conducted by Viac and Fraser (2020), higher education teachers who taught various subjects reported higher job-satisfaction when they were satisfied with autonomy in teaching decision-making and enjoyed support from leadership groups. In Latin America, Davies (2021) reported that ELT teacher satisfaction was significantly influenced by classroom resources, institutional climate, and perception of fairness in the promotion process.

Taken together, these global results suggest that while local context and education policy differ, there are certain common dimensions such as autonomy, institutional support, and professional development which have the same effect on job satisfaction for ELT professionals everywhere. Awareness of these common dimensions can inform more inclusive and responsive initiatives to support educator well-being in various higher education contexts. Moreover, job satisfaction has also been found to be strongly associated with both emotional well-being and resilience. For example, Klassen and Chiu (2010) depicted that higher job satisfaction was a predictor of decreased stress and burnout among teachers in universities and enhancing their self-efficacy. These findings point towards the need to construct positive working conditions to enable the psychological functioning of teachers.

### Teacher resilience

Beyond job satisfaction, teacher resilience has also been seen as a key construct to help us understand teachers’ ability to uphold psychological well-being and professional commitment in the face of increasing institutional pressures. More broadly, resilience is the capacity of an individual to recover from disappointments, to adapt to change, and to survive when faced with adversity (Gu and Day, 2007). In the context of education, it captures teachers’ ability to remain motivated, emotionally stable, and productive despite having been subjected to stressors like workload overload, classroom issues, and policy reform (Howard and Johnson, 2004).

Rather than a fixed trait, resilience is increasingly seen as an adaptive, developmental process influenced by dynamic interplays between individuals’ resources and environmental conditions (Mansfield et al., 2016). This is particularly important in language instruction since educators have to contend with sociocultural problems, learner diversity in needs, and continuously changing instructional demands. Such settings call for not only pedagogical ability but also resilience in regulating emotional labor and staying motivated (Mercer, 2021; Hiver, 2017). Resilience enables ELT instructors to cope effectively with hardship, transform professional trauma into learning, and retain very high degrees of purpose (Lian et al., 2025).

Several studies in regular school settings have established the significance of resilience in sustaining teacher performance and well-being. For instance, Beltman et al. (2011) concluded that resilient Australian teachers remained committed to teaching despite highly challenging classroom emotional interactions. Additionally, Day and Gu (2013) established that resilient UK teachers sustained lesson quality and emotional stability even in the face of external pressures from policies. In China, Li et al. (2024) observed university instructors with higher resilience levels exhibited less occupational stress and higher psychological well-being.

This is also evident in the Turkish school culture, where resilience has also been shown to counteract the effects of occupational stress. Koçyiğit and Sezer (2024) concluded in their research that resilient primary and secondary school teachers in Türkiye were found to have lower emotional exhaustion and were better equipped to cope with stress concerning the classroom. Furthermore, Demir Polat and İskender (2018) demonstrated that teacher resilience was a positive predictor of job satisfaction and psychological well-being, highlighting its function as an equilibrium factor in professional life, particularly in curriculum rigid systems with intensive workload.

In ELT-specific contexts, the importance of resilience is doubled. Language teachers are more likely to face higher demands, for example, performance pressure, diversity in language and culture, technical adjustment, and institutional constraints. Because of these dual forces, Hiver (2017) recommended teacher immunity, a psychological adaptation process involving resilience which helps language teachers maintain professional self and emotional well-being in the long run. Sounding the same, Fathi and Saeedian (2020) found that more resilient Iranian EFL teachers experienced greater pleasure in teaching and stronger professional identity. Similarly, Benevene et al. (2020b) found that there is a close relationship between teacher resilience and psychological wellbeing. The findings revealed that well-being mediated the relationship between climate and professional engagement for Italian teachers.

This evidence supports that teacher resilience is not strictly an individual personality variable, but a socially based and context-responsive capacity (Day and Gu, 2013; Hiver and Dörnyei, 2017). It is formed and sustained by institutional provision arrangements, collegiality, professional training, and sensed autonomy. For ELT practitioners—especially in higher education systems under stress to provide internationalization, performance, and technological demands—resilience is a psychosocial defense mechanism as well as a driver of sustainable teaching quality. Any effort, therefore, to enhance teacher resilience must move beyond individual interventions to also respond to systemic changes that allow educators to thrive despite the inherent challenges of language instruction.

### Teacher psychological well-being

Psychological well-being (PWB) is a worldwide and dimensional notion that reflects something more than the absence of stress, anxiety, or depression (Ryff and Keyes, 1995). Unlike hedonic approach identifying well-being with happiness or pleasure at a given moment, the eudaimonic perspective— conceptualized by Ryff (1989)—emphasizes the realization of personal potential and an important life. Ryff’s model outlines six interrelated aspects of well-being: autonomy (independence and self-determination), environmental mastery (competency in organizing work and life efficiently), personal growth (continuity and openness to experience), positive relationships (commitment and loving social relationships), purpose in life (goal-directness with sense of direction), and self-acceptance (positive and realistic perception of the self). They both together provide the context for analyzing how individuals subjectively experience psychological well-being in a complex and demanding world (Ryff and Keyes, 1995; Ryff, 2014).

In the academic setting, psychological well-being is particularly significant since the teaching, research, and administrative functions are emotionally as well as cognitively demanding (Benevene et al., 2020a). The college professors usually encounter role conflict, time pressure, and insubstantial institutional rewards, conditions that have the potential to undermine their satisfaction and inspiration levels (Skaalvik and Skaalvik, 2017; Benevene et al., 2020a).

Conversely, psychologically well-being instructors are more likely to use creative pedagogy, adjust to change, and favorably impact institutions (Collie et al., 2012; Viac and Fraser, 2020). Their intention to remain longer in the teaching field and their low likelihood of burnout highlight even more the connection between well-being and teacher retention.

Within ELT, psychological well-being takes the stage. ELT teachers often work in multilingual, cosmopolitan, and ever more virtual environments. Such settings demand linguistic and pedagogical competence, but also emotional competence, intercultural awareness, and adaptability (Dewaele and Li, 2020; Mercer, 2021). Moreover, teaching often involves emotional work such as challenging struggling students, diffusing tension within the class, or providing help to diverse learning needs and feelings. To that end, there is an urgency to support wellness, not just for teacher well-being but for creating caring, motivating, and emotionally safe communities of learning (Fathi and Derakhshan, 2019; King and Ng, 2018).

Importantly, teacher job satisfaction and teacher resilience have been used across all contexts as reliable predictors of teachers’ psychological well-being. Job satisfaction, which is the degree to which teachers feel valued, competent, and supported in their job, relates to emotional stability and purpose (Skaalvik and Skaalvik, 2011). Resilience acts as a buffer that helps teachers manage stress, bounce back from adversity, and stay motivated (Gu and Day, 2007). Benevene et al. (2020b) studied in the Italian university context and found that teachers who were more job-satisfied and resilient were far more likely to achieve psychological flourishing.

Despite this evidence, relatively few studies have attempted to investigate the combined effect of job satisfaction and resilience on psychological well-being in ELT contexts. This is an important gap, as Turkish ELT instructors are likely to face distinctive challenges including large teaching loads, inflexible curricula, organizational instability, and limited career progression opportunities (Valizadeh, 2021). Such pressures can disrupt satisfaction and resilience, ultimately undermining psychological well-being. Therefore, understanding how these different variables interact in the contexts of Turkish ELT is vital in facilitating evidence-based interventions and enduring, healthy teaching careers.

The purpose of this research, which is theoretically oriented towards Ryff’s multidimensional model of psychological well-being (Ryff, 1989; Ryff and Keyes, 1995), is to add to the developing field of teacher well-being by investigating the interplay of job satisfaction and resilience, and psychological well-being of ELT instructors in a higher education setting. This theoretical framework serves as a platform for exploring the presence of well-being as well as the activates that sustain well-being in academically challenging environments. These relationships will provide evidence-based rationale for the establishment of targeted interventions and institutional policies that can aid ELT teachers’ well-being which often, indirectly, will affect more broadly the quality of language teaching.

Additionally, the results have important implications for language teacher education, universities, and policy makers. By clarifying the situational determinants of well-being, including emotional resilience and job satisfaction, this research can inform strategies for establishing positive learning environments where ELT professionals feel valued, empowered, and inspired. These results can resonate with teacher retention strategies, employee performance, and ultimately student achievement, especially in the pressures of increasingly demanding and computerized environments for language teaching. With the teachers’ psychological welfare in mind, individuals inside the school structure can foster a more healthy learning climate that allows educators to positively improve the quality of teaching and therefore student outcomes.

### The present study

The literature review focuses on the fact that psychological well-being, job satisfaction, and teacher resilience are interrelated concepts that, taken together, contribute to teachers’ experiences and work outcomes. High-resilient teachers are generally more capable of handling occupational stress and are most likely to achieve greater job satisfaction and emotional stability. Conversely, inadequate institutional support and emotional resources can lead to reduced well-being and burnout. Despite growing amounts of literature on teacher resilience and job satisfaction, their combined effect on psychological wellbeing, particularly in ELT context in higher education, is not well researched.

Several gaps were identified in current literature that motivated the development of the present study. Firstly, much of previous research has focused on measures of burnout, or job performance, rather than well-being as a positive psychology construct based upon emotional health and satisfaction (Mercer, 2021). Second, the majority of these studies have been conducted in countries with a similar infrastructure, with little evidence from other contexts with diverse social and institutional conditions like Türkiye, where contextual, institutional, and cultural forces may shape teachers’ experiences differently. Lastly, even though teacher happiness has more and more become the subject of conversation in the broader education literature, it remains slightly under-explored in ELT, particularly within the sphere of universities (MacIntyre et al., 2020; Mercer, 2020; Hofstadler et al., 2021; Sulis et al., 2024).

To address these gaps, the present study focuses on university-level ELT instructors in Türkiye and investigates the relationships among job satisfaction, teacher resilience, and psychological well-being. Specifically, the study addresses the following research questions:

1. How are job satisfaction, teacher resilience, and psychological well-being related among ELT university instructors in Türkiye?
2. To what extent do job satisfaction and teacher resilience predict the psychological well-being of ELT university instructors?

**Figure 1:**
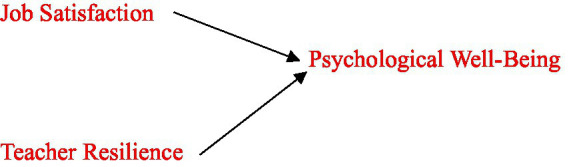
Hypothesized model showing the predictors of psychological well-being among ELT university instructors.

Based on the theoretical framework (Ryff, 1989; Ryff and Keyes, 1995) and current empirical research, this study hypothesizes that job satisfaction and teacher resilience are important predictors of psychological well-being among university-level ELT instructors. Together, these constructs are thought to impact teachers’ general well-being in their professional setting. The hypothesized model is depicted in Figure 1.

## Methods

### Participants and the study context

The sample of the study comprised 173 university-level ELT instructors, of whom 65 were males and 108 were females, aged between 25 and 55 years (M = 38.6, SD = 6.5). The participants’ years of teaching experience ranged from 2 to 30 years. Regarding educational qualifications, 110 participants held a BA in English Language Teaching (ELT), 50 held an MA degree, and 13 held a Ph.D. The participants signed informed consent before being admitted to the study. Overall, the study encompassed a diverse and heterogeneous population of language instructors, such as participants with different educational and experience backgrounds. Regarding the study context, English is widely taught as a mandatory foreign language course and, in most cases, as a medium of instruction, particularly in departments that offer programs in English. Most universities, especially state and foundation (private) ones, require students to meet a specified level in an English proficiency test before enrolling in department courses. Students who do not meet the minimum required level are typically directed to a one-year preparatory program in English, during which they receive intensive training in academic reading, writing, listening, and speaking skills [Council of Higher Education (YÖK), 2020]. In English Language Teaching (ELT), English Translation and Interpretation, and English Language and Literature departments, students are meant to learn English at an advanced level throughout their study. However, challenges such as varying levels of student competence, limited classroom communication, and uneven incorporation of communicative activities continue to impact the effectiveness of English language teaching at the higher education level (British Council and TEPAV, 2015; Kırkgöz and Dikilitaş, 2018).

## Instruments

### Teacher job satisfaction questionnaire (TJSQ)

Teacher job satisfaction was measured using an adapted version of the Teacher Job Satisfaction Questionnaire (TJSQ), originally developed by Lester (1987) and then shortened into a 24-item short form by Almutairi (2020). However, the Salary subscale was not included in the present study due to its limited relevance in the context of higher education in Türkiye, where salary structures are standardized and centrally regulated. The items were directly adopted from Almutairi’s validated 24-item short form and only the salary items were removed for context, yielding a final count of 21 items.

Sample items included: *“My supervisor help me with improving my teaching performance,” “I receive helpful feedback and suggestions from my colleagues,” “I have opportunities to get professional development,”* and *“I feel safe in my present job as a teacher.”* Each item was rated on a 5-point Likert scale from 1 (strongly disagree) to 5 (strongly agree), with higher scores indicating greater job satisfaction levels. The TJSQ was administered in its original English form since the participants were ELT instructors and have high proficiency levels in English, and no translation or linguistic administration was needed.

The adapted scale exhibited high internal consistency (Cronbach’s *α* = 0.84), indicating that it possessed high reliability. This result aligns with Almutairi’s (2020) findings in which he reported high value reliabilities (range *α* = 0.77–*α* = 0.94) across multiple educational contexts.

Overall, the findings indicate that the 21-item adapted TJSQ is a valid and reliable instrument for measuring job satisfaction for ELT instructors within higher education institutions in Türkiye.

### Teacher resilience scale

Teacher resilience was evaluated using the Connor-Davidson Resilience Scale (CD-RISC) developed by Connor and Davidson (2003), which is a valid and reliable self-report instrument that evaluates people’s ability to cope with adversities and sustain psychological health. The original scale is a 25-item measure, with each item scored on a five-point Likert-type scale, ranging from 0 (not true at all) to 4 (true nearly all the time). The CD-RISC is conceptualized as a multidimensional construct in time, and it assesses a variety of mental health constructs, such as personal competence, high standards, and perseverance, trust in one’s instincts and tolerance of negative affect, positive acceptance of change and secure relationships, control, and spirituality (Connor and Davidson, 2003). Psychometric research has shown that CD-RISC has a high degree of internal consistency, with Cronbach’s alpha usually >0.89 (Connor and Davidson, 2003). In this current study, the CD-RISC also showed good reliability; that is, it produced a Cronbach’s alpha coefficient of 0.92 suggesting an excellent level of internal consistency in ELT instructors. The CD-RISC has been shown to have construct validity across many populations including education with confirmatory and exploratory factor analyses, and test–retest reliability coefficients confirm it has good temporal stability, making it a robust measure of resilience.

In the context of ELT, the CD-RISC provides us with a snapshot of the teacher’s psychological resources that enable them to deal with multiple challenges related to language education. For example, items such as “*I can deal with whatever comes my way”* or *“I tend to bounce back after illness or hardship*” highlight the essential qualities of resilience that ELT instructors utilize regularly when contextualizing their role in ELT context, high expectations, institutional limitations, or classroom nonlinearity. Remaining calm under pressure, adapting their style to ever-changing curricula, and motivating themselves with limited resources are essential competencies that the CD-RISC identifies in the ELT area. Mercer (2020) states that ELT professionals experience forms of emotional labor, multi-ethnic classrooms, and teaching environments that apply stress in different forms, which require resilience in order to promote their professional well-being and effectiveness. Overall, a measure of resilience such as the CD-RISC provide advantages for those who work with teachers, as knowing that resilience can be measured can provide avenues for interventions to promote resilience, and consequently performance, teacher retention, and job satisfaction (Campbell-Sills and Stein, 2007).

### Psychological well-being scale—short form (PWB-SF)

The short version of Ryff (1989) Psychological Well-Being Scale was used as the multi-dimensional measure for psychological well-being. This scale uses a eudemonic model, which assesses six components of optimal psychological functioning (subscales): autonomy, environmental mastery, personal growth, positive relations with others, purpose in life, and self-acceptance. The brief 18-item scale used in this study utilized three items from each subscale, rated on a 6-point Likert type scale from 1 (strongly disagree) to 6 (strongly agree). The higher scores indicate the better levels of psychological well-being across the dimensions. This concise format is especially beneficial for survey research, where it is essential to optimize brevity in questions while preserving high psychometric quality. It has been used on a variety of international samples, having an adequate internal consistency, with their Cronbach’s alpha score (*α*) for subscales usually being in the range of *α* = 0.70 to 0.85 (Ryff and Keyes, 1995). The reliability in this study was also satisfactory with a Cronbach’s alpha of 0.81. This suggests that it is suitable for use in the ELT higher education context.

At the university level, there are characteristics of the dimensions emphasized on this scale that have considerable significance. ELT instructors are likely to engage in affectively laden tasks which require a combination of environmental mastery and personal growth such as *In general, I feel I am in charge of the situation in which I live* or *I think it is important to have new experiences that challenge how you think about yourself and the world.* Similarly, developing autonomy and purpose in life is paramount to maintaining the internal motivation to teach, especially considering all the organizational constraints, which are depicted in the items *I judge myself by what I think is important, not by the values of what others think is important* and *For me, life has been a continuous process of learning, changing, and growth*. Positive relations demonstrate the value placed on developing intercultural and collegial communicative competence, while self-acceptance and growth reflect long-term accommodation and reflection in a rapidly developing profession immersed with learner diversity.

### Procedures and data analysis

The research on teachers’ job satisfaction, resilience, and psychological well-being in the ELT context was conducted using an online questionnaire. Participants were invited through institutional mailing lists, academic networks, and professional social media platforms commonly used by ELT instructors in Türkiye. The survey link included informed consent and confidentiality assurances. A total of 173 instructors out of 240 responded, meaning that the response rate was a respectable 72%, which for an online questionnaire is quite good. This should mean that participants were probably well-motivated and involved, and possibly a fair representation of the broader community of ELT instructors in Turkish higher education. However, it is worth noting that there is potential for self-selection bias since people who willingly answer such studies may vary in terms of motivation, professional fulfillment, or psychological attitude from those who choose not to respond.

Data gathered were treated with Pearson correlation and hierarchical multiple regression analysis in order to study the strength and direction of association between the core variables. Hierarchical regression was used to assess the incremental contribution of job satisfaction and resilience in predicting psychological well-being (Pallant, 2016). In other words, incremental contribution represents the additional variance explained and attributed to well-being by each predictor included in the model. This method provided a clearer image of the relative impact of resilience and job satisfaction in deciding well-being and allowed mediating effects to be detected.

## Results

Based on the descriptive statistics, instructors’ reported job satisfaction levels (M = 3.32, SD = 0.94) and their resilience scores (M = 3.03, SD = 0.68) were moderate while their psychological well-being levels (M = 4.15, SD = 0.87) were somewhat high. The distributional characteristics of the study variables were assessed for normality using skewness and kurtosis statistics. The values of skewness for job satisfaction (0.034), resilience (−0.038), and psychological well-being (−0.097) were all close to zero, which implied that the distributions of the data are nearly symmetric and not much different from the normal distribution. Values of skewness that fall within ±1 can be accepted for assuming normality for the purposes of psychological, educational, and related research (George and Mallery, 2019). With respect to kurtosis, the excess kurtosis for job satisfaction (0.580), resilience (0.562), and psychological well-being (−0.315) were all well within the conventional bound of ±1, indicating that they are mesokurtic and have similar peakness and thickness of tails to the normal distribution (West et al., 1995). Taken together, these results indicated that the variables are approximating normality, satisfying the assumptions for parametric statistics that are commonly undertaken in social sciences research (Tabachnick and Fidell, 2019) (Table 1).

**Table 1:** Descriptive statistics.

Variables	*N*	Mean	Std. deviation
Job satisfaction	173	3.32	0.94
Resilience	173	3.03	0.68
Psychological well-being	173	4.15	0.87

Regression analyses provided an important overview of the use of predictors (job satisfaction and teacher resilience) and the dependent variable (psychological well-being) (Table 2). There were statistically significant positive correlations between job satisfaction and teacher resilience (r = 0.472, *p* < 0.01), job satisfaction and psychological well-being (r = 0.632, *p* < 0.01), and resilience and psychological well-being (r = 0.502, *p* < 0.01). Overall, it appears that job satisfaction and resilience among ELT instructors are positively associated with their psychological well-being (see Table 3). For these analyses, two models were examined to assess the impact of predictors on psychological well-being.

**Table 2:** Correlations among psychological well-being, job satisfaction and teacher resilience.

Variables	1	2	3
1. Psychological well-being	1.000		
2. Job satisfaction	0.632	1.000	
3. Teacher Resilience	0.502	0.472	1.000

*N* = 173. *p < 0.05 (two-tailed).

**Table 3:** Model summary.

Model	R	R square	Adjusted R square	Std. error of the estimate	Change statistics				
					R square change	F change	df1	df2	Sig. F change
1	0.632	0.382	0.374	6.015	0.382	63.762	1	120	0.000
2	0.674	0.431	0.420	5.737	0.049	8.253	1	119	0.002

Model 1 which only included job satisfaction as a predictor explained a statistically significant variance in psychological well-being (R^2^ = 0.382, adjusted R^2^ = 0.374, *p* < 0.001), and the relationship between job satisfaction and psychological well-being was moderate and positive (R = 0.632).

Model 2 was the expansion form of Model 1, but additionally included teacher resilience as well as job satisfaction as predictors. Model 2 was a better fit that accounted for more variance in psychological well-being (R^2^ = 0.382, adjusted R^2^ = 0.431, *p* < 0.001). The correlation in Model 2 (R = 0.674) indicated a stronger positive relationship between the predictor variables and psychological well-being than in Model 1. The F statistic for both models was also significant, which indicated that the model as a whole ability to predict the outcome variable, psychological well-being, was significant [Model 1: *F*_(1, 120)_ = 63.762, *p* < 0.001; Model 2: *F*_(2, 119)_ = 8.253, *p* < 0.001]. Finally, the change in R^2^ between the two models was significant [ΔR^2^ = 0.049, Δ*F*_(1, 119)_ = 8.253, *p* < 0.001], which indicated that the variance in psychological well-being explained by the model improved with the addition of teacher resilience as an additional predictor for the study.

Table 4 presents the results of an analysis of variance (ANOVA) to determine the significance of the predictors in the linear regression model. The ANOVA indicated both the regression models significantly predicted psychological well-being. In Model 1, the predictor job satisfaction was significantly related to psychological well-being [*F*_(1, 120)_ = 63.762, *p* < 0.001]. This model explained a substantial amount of the variance in psychological well-being. In Model 2, which includes teacher resilience as a predictor, the model was significant with psychological well-being [*F*_(2, 119)_ = 38.574, *p* < 0.001]. In other words, the addition of the these predictors explained a significant improvent in the model.

**Table 4:** ANOVA results.

Model		Sum of squares	df	Mean square	F	Sig.
1	Regression	3283.74	1	3283.74	63.762	0.000
	Residual	6716.26	120	51.50		
	Total	10000.00	121			
2	Regression	3688.84	2	1844.42	38.574	0.000
	Residual	6311.16	119	47.82		
	Total	10000.00	121			

Table 5 provides the results for the model coefficients. The regression coefficients are essential in determining the relationship between the predictors. In Model 1, job satisfaction was a significant predictor of psychological well-being (*β* = 0.632, *p* < 0.001), meaning the higher the job satisfaction, the greater psychological well-being. In Model 2, the job satisfaction coefficient was still significant (*β* = 0.512, *p* < 0.001), and teacher resilience became a significant predictor of psychological well-being (*β* = 0.265, *p* < 0.001) indicating both teacher job satisfaction and resilience were significantly related to psychological well-being.

**Table 5:** Multiple regression analysis.

Model	Predictor	B	Std. error	Beta	*t*	Sig.
1	(Constant)	52.35	5.20		10.075	0.000
	Job satisfaction	0.678	0.51	0.632	8.050	0.000
2	(Constant)	45.70	6.10		6.752	0.000
	Job satisfaction	0.526	0.58	0.512	5.873	
	Teacher resilience	0.351	0.73	0.265	3.154	0.000

The relationships between the predictors and dependent variable were consistent with the standardized coefficients. Job satisfaction indicated a strong positive relationship with psychological well-being (r = 0.632, *p* < 0.001), while teacher resilience exhibited a moderate positive relationship (r = 0.502, *p* < 0.001).

In summary, the findings of the present study indicated that both job satisfaction and teacher resilience were significant predictors of psychological well-being among ELT university instructors. Model 1 revealed that job satisfaction predicted 37.4% of psychological well-being variance. On the other hand, Model 2 indicated that the addition of teacher resilience in responsiveness to job satisfaction, raised the adjusted R^2^ from 37.4% of variance attributed to job satisfaction to 42%. This indicated that teacher resilience improved the explanatory power of the model by roughly 4.6%. Therefore, it can be concluded that organizational factors along with personal psychological resources contribute to the psychological well-being outcomes of ELT instructors in Turkish higher education contexts.

## Discussion

Given the establishment of teacher well-being as a priority, within higher education, this study examined the relationship between university-level ELT instructors’ job satisfaction, resilience and psychological well-being within the Turkish context, and also its possible question to a larger extent of which of these variables acted as a stronger predictor of psychological well-being. Data were collected from 173 ELT instructors via validated self-report questionnaires. Findings indicated that psychological well-being was positively correlated with, and showed significant association with, both job satisfaction and resilience. Once again, those instructors with higher satisfaction with their role and those with higher resilience had higher reported psychological well-being.

Related to the first research question, the findings were consistent with other studies that emphasize an institutional support and individual psychological resources are important when predicting teacher well-being (Mercer, 2021; Viac and Fraser, 2020). The strong positive relationship between job satisfaction and psychological well-being provides evidence that enhancing teachers’ job satisfaction is a primary factor in teachers’ overall psychological well-being. This finding also aligns with literature that suggests teachers who view their working environment as fulfilling and supportive will experience higher levels of mental and emotional health (e.g., Skaalvik and Skaalvik, 2017). Moreover, it emphasizes the need for organisational practices and policies to enhance job satisfaction as a proactive way of protecting teachers’ psychological resources and ongoing well-being.

In the ELT context specifically, where teachers often work in linguistically and culturally diverse classrooms and constant pressures from internationalization and digital transformation, strong resilience is essential (King and Ng, 2018; Hiver, 2017). Likewise, job satisfaction emerged as a significant correlate of well-being, corroborating previous research that educators who feel valued, able to practice professional autonomy, and supported by leadership are more likely to report a sense of fulfillment and mental stability (Skaalvik and Skaalvik, 2011; Klassen and Chiu, 2010). In language education, job satisfaction will be impacted by on-going professional development opportunities, acknowledging their linguistic expertise, and their role in curriculum design (Mercer and Gregersen, 2020). Because language teaching suggests more cognitive and emotional labour (e.g., scaffolding communication, managing affective filters, promoting intercultural sensitivity), job satisfaction offers teachers not only a protective factor against professional burnout but even a precondition for pedagogical creativity (Gkonou et al., 2020).

To investigate the relative predictive strength of job satisfaction and resilience on psychological wellbeing, hierarchical regression analysis was conducted in response to the second research question. The analysis indicated that both job satisfaction and resilience were significant predictors of psychological wellbeing, however, job satisfaction was a stronger predictor and was able to add more explanatory power to the model. This finding is consistent with previous research by Mercer and Gregersen (2020) who emphasized the importance of satisfaction in fostering sustained professional motivation and emotional health throughout a teacher’s career. The results are aligned with Self-Determination Theory as suggested by Ryan and Deci (2017) that individuals with a sense of competence are more likely to have intrinsic motivation, being engaged in their work ultimately contributes to psychological well-being. In ELT, varying needs of students are the norm (Derakhshan et al., 2022), resiliency is a useful tool for teachers as they regularly juggle stress while providing quality instruction in an uncertain scenario.

Finally, the study highlights the complexity and interplay of teacher psychological well-being in ELT. Teacher well-being was seen as being influenced by both not just one factor, but more so, external conditions (e.g., support from an institution, collegiality) and internal coping resources (e.g., emotional regulation, change ability). This supports the view of teacher well-being as multifaceted and reliant on the contextual and personal factors (Day and Gu, 2013; Mansfield et al., 2016). For ELT practitioners specifically, the practice of teaching a second or foreign language is often complicated by the requirement to inhabit multiple roles in an exam-driven, multilingual, and/or technology-enhanced context. As a result, supporting teacher well-being will require a collective response that attempts to do more than support a single individual to attend to their well-being (Gkonou et al., 2020).

Given these results, there are implications for policymakers, teacher educators, and university leaders. Firstly, we need to pay attention to supporting resilience building in instructors. This could take the form of targeted interventions, such as coaching schemes and opportunities to engage in reflective practice and collaboration with peers that help instructors develop emotional regulation, perseverance and adaptive coping skills. In the ELT context, that might involve creating professional learning communities (PLCs) focused on intercultural pedagogy, digital mediation, and student engagement. It is worth noting that Hiver (2017) previous work on teacher immunity lends support to the notion that resilience-building in this way is part of the minute-by-minute work of sustained and successful professionals.

Secondly, we should promote job satisfaction through better working conditions. Institutions can enact positive changes by doing things such as sharing workloads more fairly, recognizing teachers’ efforts, increasing professional development options, and increasing collegial relationships. The above practices have all been shown to create a more supportive work environment and have led to increased overall job satisfaction (OECD, 2021). For ELT teaching professionals, providing opportunities for teacher voice in decisions related to assessment practices, textbook selection, or digital tools could in a way enhance their perceived autonomy and value. These various levels of professional responses should improve instructor wellbeing and ultimately influence teaching quality and student learning, along with advancing the common goals of educational excellence in higher education.

Third, as there is evidence that psychological well-being can impact student learning and achievement (Collie et al., 2015; Sulis et al., 2024) by influencing emotional climate, investments that promote teacher well-being can maintain backing for institutional effectiveness. ELT instructors who are resilient and satisfied will be more able to foster a positive classroom, model adaptive behaviors, and promote student engagement and success (Mercer and Dörnyei, 2020). In particular, this situation applies to EFL contexts like Türkiye where motivation and retention have always posed challenges to university-level English instruction.

Overall, the research provides support for the importance of both job satisfaction and resilience in the well-being of ELT teachers working in higher education. However, the role of job satisfaction was marginally more predictive, indicating that fostering ELT teachers’ psychological coping mechanisms may be the best investment for effective well-being and professional sustainability. Subsequent research may expand on this model by including other psychological variables, such as teacher autonomy, perceived competence, or institutional trust with the aspiration of understanding the well-being ecology of ELT contexts.

## Conclusion

This study was conducted to assess the psychological wellbeing of ELT university instructors in Türkiye, but also sought to identify the predictive power of job satisfaction and teacher resilience. It was shown that job satisfaction and teacher resilience, both predicted ELT university instructors’ psychological wellbeing, with job satisfaction as the better predictor. These results demonstrate that an interrelated nature exists between institutional characteristics and individual psychological characteristics to influence teachers’ wellbeing in higher education contexts.

The study supports the view that in order to enhance the psychological well-being of ELT instructors is important not only for their own professional sustainability but for them to provide quality instruction and a productive learning environment. Instructors that believe they are effective, are supported and feel emotionally capable are more likely to stay invested and engaged or to rebound from circumstances that challenge their work ability and help their students to succeed. Thus, the study revealed a collective responsibility on everyone involved in preparing the next generation of educators, as well as decision makers in universities and people who make educational policy, to create a place for work that is conducive to both professional satisfaction and emotional resilience.

Teacher educators can assist by bringing wellbeing- focused modules and reflective practices into teacher education programs, scaffolding pre-service and in-service teachers in finding coping mechanisms for their mental well-being (as well as opportunities for reflective thinking about themselves). In support of this, university administrators should recognize some of the organizational structures that help foster this type of well-being, such as consideration of manageable workloads, effective recognition systems, collaborative professional learning groups, and a willingness to bridge the space between academia and the classroom. Lastly, on a policy level, there is a need to create systemic structures and frameworks for career development, teacher choice and agency, and equitable working conditions in the area of language education.

Although the study did make some useful contributions, there are some limitations. First, although the sample size (N = 173) offered a reasonable sample, it may not capture the full diversity of the Turkish ELT higher education context. Future research might include the use of a sample that is more widely distributed in institutional types, geographical regions, and experience to enhance generalizability. Second, while best effort was made to triangulate data collection method (survey), the study still used self-report, which can be subject to both social desirability and subjective interpretation. Future research employing any combination of qualitative methods (e.g., interviews, focus groups, teachers’ narratives) might provide richer understandings and perceptions regarding the lived experiences of instructors. Finally, because data was collected at only one time-point, causality is ambiguous. While we report correlations between variables, the study only captured relationships and directionality among variables.

Future research with longitudinal and experimental designs would provide a stronger evidence base for how job and resilience interact over time to contribute to psychological well-being.

In summary, this study extends the research base on teacher well-being in language education by identifying key psychological and organizational predictors of well-being for university-level ELT instructors. The results highlighted the need for institutional and systemic support for educational resilience and job satisfaction, which will contribute to more sustainable teaching in increasingly demanding and digital academic environments.

## Data Availability

The original contributions presented in the study are included in the article/supplementary material, further inquiries can be directed to the corresponding author.
